# Critical stages in pea photosynthesis impaired by tetracycline as an environmental contaminant

**DOI:** 10.1007/s10265-024-01580-x

**Published:** 2024-09-21

**Authors:** Magdalena Krupka, Dariusz J. Michalczyk, Agnieszka I. Piotrowicz-Cieślak

**Affiliations:** Department of Plant Physiology, Genetics and Biotechnology, University of Warmia and Mazury in Olsztyn, Olsztyn, Poland

**Keywords:** Pea, Ribulose bisphosphate carboxylase-oxygenase activity, Tetracycline

## Abstract

The widespread use of antibiotics in intensive animal husbandry, and the agricultural utilization of manure from such farms, imposes a significant burden on the environment. Consequently, the effects of antibiotics should be studied not only in animals and humans but also in all components of biocenoses and agrocenoses. In our study, we analyze the impact of four different concentrations of tetracycline present in soil (0, 5, 50, and 500 mg/kg of soil) on the growth and key photosynthesis parameters of pea seedlings: chlorophyll concentration, aminolevulinic acid concentration, aminolevulinic acid dehydrogenase activity, and ribulose bisphosphate carboxylase-oxygenase (RuBisCO) activity. At the lowest tetracycline concentration, chlorophyll content decreased by 13% compared to the control (0 tetracycline), while at the highest antibiotic concentration, it decreased by as much as 27%. Similarly, the decrease in aminolevulinic acid (a chlorophyll precursor) concentration was significant, amounting to 34%. However, the activity of the dehydrogenase enzyme, which consumes this precursor, decreased even more drastically by 51%, indicating significant disturbances in the light phase of photosynthesis. However, the activity of RuBisCO in pea plants subjected to tetracycline was even more severely affected, dropping by 58%, 69%, and 70% in soils with increasing concentrations of tetracycline. The reduction in enzyme activity could only partially be explained by a less pronounced decrease in the quantity of RuBisCO (large subunit) protein, which amounted to 6.5%, 11%, and 35% for tetracycline concentrations of 5, 50, and 500 mg/kg of soil, respectively.

**Supplementary Information:**

The online version contains supplementary material available at 10.1007/s10265-024-01580-x.

## Introduction

Soil pollution is a serious environmental issue. Recently, antibiotics and their metabolites have been detected in soils. The widespread use of pharmaceuticals in animal production is one of the main causes of the entry of these compounds into the environment (Gržinić et al. 2023). In intensive and mass animal production, antibiotics are used for therapeutic, preventive, and growth promotion purposes (Muaz et al. 2018). Although the use of antibiotics as growth promoters has been banned in EU countries, they are still administered therapeutically to the entire herds instead of individual animals (FAO 2021). In Asian countries, there are legal regulations regarding the use of antibiotics in animal production, but these substances are still widely available and used, among others as growth promoters (Shao et al. 2021). According to the CDEEP report, the annual consumption of antibiotics by livestock will exceed 200,000 tons by 2030. The incomplete metabolism of these drugs in animal bodies results in the excretion of approximately 40–90% of the administered dose in an active form in urine and feces (Polianciuc et al. 2020). Tetracycline (TC) is one of the most commonly used antibiotics in animal production, and its residues are found in agricultural soils worldwide (Conde-Cid et al. 2020). The application of untreated manure to fields containing tetracycline residues is one of the main sources of this antibiotic in soil. The lack of effective methods for removing antibiotics in wastewater treatment plants means that irrigation of fields with processed waste water may also lead to the antibiotics entering soil. Tetracycline present in the soil is taken up and accumulated in plant tissues (Tasho and Cho 2016), causing morphological and biochemical changes (Rocha et al. 2021).

Numerous studies indicate that photosynthesis is the most sensitive metabolic process to the action of antibiotics (Krupka et al. 2022). Antibiotics can enter chloroplasts, possibly with the help of MAR1 transporters (Conte and Lloyd 2010; Margas et al. 2019), thereby directly affecting the photosynthetic apparatus. The first visible symptoms of damage to the photosynthetic apparatus include leaf yellowing (Margas et al. 2019; Rydzyński et al. 2017). At the biochemical level, the effects of antibiotics on the photosynthetic apparatus are not yet fully understood. Several studies suggest that one of the causes of damage to the photosynthetic apparatus may be the accumulation of reactive oxygen species (ROS) in chloroplasts. Hájková et al. 2019 suggested that a 10-minute exposure to pharmaceuticals results in an increase in ROS content in chloroplasts. The accumulation of ROS then leads to lipid peroxidation in thylakoid membranes (Hájková et al. 2019). Proteins associated with photosystem II are particularly sensitive to ROS accumulation (Gomes et al. 2020). A detailed characterization of photosystem damage under the influence of antibiotics is presented in Krupka et al. (2021).

Tetracyclines inhibit bacterial protein synthesis by binding to the bacterial 30 S ribosome subunit (Scaria et al. 2021). Some studies suggest that antibiotics that block bacterial protein biosynthesis are particularly toxic to the photosynthetic apparatus (Liu et al. 2011) due to the fact that chloroplasts have bacterial transcription and translation mechanisms (Kasai et al. 2004). In addition to directly affecting the proteins involved in photosystems, antibiotics likely also affect the chlorophyll biosynthesis pathway (Krupka et al. 2022). The chlorophyll biosynthesis pathway involves 16 reactions catalyzed by 16 enzymes encoded by more than 20 different genes (Beale 2005). Chloroplast protein complexes are encoded by both nuclear and chloroplast genes (Yoon et al. 2020). Koussevitzky et al. (2007) demonstrated that the expression of a nuclear gene involved in chlorophyll synthesis can become inhibited when the chloroplast gene expression is not occurring. Therefore, antibiotics can affect chlorophyll biosynthesis at the very beginning of the biosynthetic pathway once they enter the chloroplast. However, most studies neglect this aspect. Another aspect of the impact of antibiotics on photosynthesis is their effect on the dark phase of photosynthesis. Ribulose-1,5-bisphosphate carboxylase/oxygenase (RuBisCO) is a key enzyme of the Calvin cycle, catalyzing the binding of CO_2_ to ribulose-1,5-bisphosphate (RuBP). The activity of this enzyme is a key factor determining the efficiency of photosynthesis. Margas et al. (2019) demonstrated a decrease in RuBisCO content in plants exposed to tetracycline, although its activity in plants treated with tetracycline has not yet been investigated. Most studies on the impact of antibiotics on photosynthesis are speculative in nature, and interactions between antibiotics and the photosynthetic apparatus are still not fully understood. Most of the available studies on antibiotic interactions in the photosynthetic apparatus refer to chlorophyll degradation and consequent visible changes at the morphological level. Leaf yellowing is a visible end-effects of tetracycline-induced damage, but does not provide information on the molecular and biochemical mechanisms leading to these changes. The mechanism of tetracycline-induced chlorophyll degradation has already been studied (Rydzyński et al. 2019). However, to fully understand the effects of tetracycline on the photosynthetic apparatus, it is necessary to identify the exact sites of tetracycline action in plants, such as chlorophyll biosynthesis and the Calvin cycle. Therefore, the aim of this study is to assess how tetracycline affects key biochemical and molecular elements of the photosynthetic process of peas (*Pisum sativum* L. cv. Cysterski) growing in soil contaminated with tetracycline at concentrations of 5, 50, and 500 mg/kg. Additionally, the utility of spectrophotometric methodology for assessing the activity of RuBisCO in pea seedlings was evaluated. To the best of our knowledge, such studies are rare in the literature. Photosynthetic parameters such as chlorophyll precursor content, chlorophyll biosynthetic pathway enzyme activity and RuBisCO enzyme activity will allow a multi-stage evaluation of the effect of tetracycline on the photosynthetic apparatus of plants. This will allow identification of the key steps at which tetracycline interferes with photosynthesis and an understanding how this interference leads to the negative effects observed in plants. Tetracyclines are antibiotics routinely detected in agricultural soils. Their concentrations in soils usually range from 5 to 25 mg/kg (Zheng et al. 2020), whereas in manure, concentrations exceed 700 mg/kg (Santás-Miguel et al. 2022). Tetracycline is a poorly degraded antibiotic that is constantly released into the environment (Amangelsin et al. 2023), so higher concentrations were also used in the study.

## Materials and methods

### Pea growth conditions

200 g of soil was added to 9 cm × 9 cm pots. Tetracycline at concentrations of 5, 50 and 500 mg/kg was added to pots. Tetracycline hydrochloride was added as an aqueous solution in 15 ml distilled water according to Margas et al. (2019). The control group were pots without tetracycline watered with 15 ml distilled water. Four pea seeds were placed in each pot immediately after the addition of the antibiotics. Plants were grown in a greenhouse at 23 °C/16°C day/night, 16/8 h photoperiod in 8 klx light intensity. After 3 weeks the plants were harvested for further analysis.

### Pea growth analysis

Pea growth analysis included germination (%), root length (cm), stem length (cm) and leaf area (cm^2^). For the germination test, the seeds were surface sterilized with a 1% sodium hypochlorite solution for 10 min and then thoroughly rinsed with distilled water. 10 seeds were placed in pots and transferred to the greenhouse with 23 °C/16°C day/night temperature in 8 klx light intensity. Seeds were considered germinated if the root reached a length of at least 2 mm. For root and stem length, after 3 weeks of growth, plants were gently pulled from the soil and rinsed with water. The roots were straightened without damaging them and their length was measured with a ruler to 1 mm accuracy. Stems were measured from the base (the point where the root turns into a stem) to the apex, using a ruler to 1 mm accuracy. The leaf area was determined using a VHX-7000 digital microscope equipped with image analysis software (Keyence, Osaka, Japan).

### Isolation and measurement of chlorophyll absorption

Chlorophyll was isolated according to the method of Rydzyński et al. (2017) with minor modifications. 500 mg of pea leaves were homogenized with a pestle and mortar in 5 ml of methanol (Fisher Chemicals). The homogenate was centrifuged at 1500 × *g*. The resulting supernatant was diluted 6 − fold with methanol. The absorption spectra of solutions were measured using TECAN Infinite 200 PRO. Chlorophyll a, b and total chlorophyll concentration was calculated according to Porra et al. (1989). Additionally, calculations were performed based on the absorption at λ = 664 nm according to the Lambert-Beer law, using the molar extinction coefficient of 66,600 M/cm for chlorophyll in methanol according to Seely and Jensen (1965).

### Aminolevulinic acid dehydrogenase (ALAD) activity

ALAD was extracted according to the method of Jiao et al. (2015) with minor modifications. 500 mg of pea leaves was ground in liquid nitrogen in a cold mortar and 5 ml of a mixture containing 0.05 M Tris-HCl pH 8.2 and 0.1 mM DTT (Sigma − Aldrich, USA). The homogenate was filtered through gauze, then centrifuged at 10,000 × *g* for 30 min at 4°C and the supernatant was taken. 0.27 ml ALA (concentration 1 mg/ml), 1.35 ml 0.05 M Tris-HCl pH 8.2 with 0.1 mM DDT, 0.08 ml 0.2 M MgCl_2_ were added to 1 ml of the supernatant,. The mixture was incubated at 37 °C for 1 h. The reaction was stopped by adding 0.3 ml of 3 M trichloroacetic acid (TCA). After cooling, the samples were centrifuged at 2000 × *g* for 10 min. 1 ml of Ehrlich’s reagent (4-(dimethylamino) benzaldehyde, Sigma − Aldrich, USA) was added to 1 ml of the supernatant After 10 min, the absorbance was measured (Cecil spectrophotometer, CE2021 2000 Series) at λ = 555 nm. ALAD activity was expressed as the amount of porphobilinogen (PBG) formed per 1 min per mg of protein per cm^2^ of leaves. The molar extinction coefficient of 6.2 × 10^4^ M/cm was used for the calculations according to Kumar and Charan (1998).

### Aminolevulinic acid (ALA) content

ALA content was determined according to the method of Jiao et al. (2015). 500 mg of pea leaves were ground in liquid nitrogen in a cold mortar and 3 ml of acetate buffer, pH 4.6. The homogenate was centrifuged at 10,000 × *g* for 20 min at 4°C. Then, 4 drops of ethyl acetate (Chempur) were added to 1 ml of the supernatant and boiled for 15 min in a water bath. After cooling, 4 ml of Ehrlich’s reagent (4-(dimethylamino)benzaldehyde, Sigma − Aldrich, USA) was added to the samples. After 15 min, the absorbance was measured at λ = 533 nm (Cecil spectrophotometer, CE2021 2000 Series). ALA concentration was calculated according to Averina et al. (2010), using the molar extinction coefficient for ALA 6.8 × 10^4^ M/cm. ALA concentration was expressed in nmol per cm^2^ of leaves.

### Leaf protein isolation and SDS-PAGE electrophoresis

Leaf protein isolation was carried out according to Li et al. (2009) with minor modifications. 500 mg of pea leaves were homogenized in liquid nitrogen with mortar and pestle. Then, 3 ml of isolation buffer containing 5 mM β − mercaptoethanol (Sigma – Aldrich, USA), 50 mM Tris – HCl (pH 8) and 12.5% (v/v) glycerol (Chempur) was added. The samples where then centrifuged at 1500 × *g* for 15 min at 4 °C. Protein concentration in the resulting supernatant was carried out using Bradford assay (1976) using bovine serum albumin (Sigma − Aldrich, USA) as a standard.

Resulting supernatants were then mixed with dissolving solution containing 2% SDS (w/v), 4% β – mercaptoethanol (v/v) and 10% glycerol (v/v), so as to obtain the final protein concentration of 2 mg/ml. The samples were incubated in a dry bath for 5 min at 100 °C. The samples were cooled down and 40 µg of proteins were loaded to a 10% separating and 5% concentrated polyacrylamide gel (7.0 × 10.0 cm). SDS-PAGE electrophoresis (Mini PROTEAN TetraSystem; Bio-Rad) was run at 80 V for 10 min and 140 V for 1 h. After electrophoresis the gels were stained in 0.25% Coomassie Brilliant Blue G-250 (Sigma − Aldrich, USA) solution for 12 h. The gels were destained until the background was colourless. Gel images were digitized with Gel Doc EZ Imager (Bio-Rad). Electrophorograms were analyzed and RuBisCO large subunit concentration was calculated with ImageLab software (Bio − Rad) using peaks intensity, corresponding to indentified proteins. The identification of RuBisCO large subunit was carried out using commercial RuBisCO (Sigma − Aldrich, USA). The commercial RuBisCO was prepared in the same way as proteins samples using dissolving solution and loaded to a polyacrylamide gel.

### Isolation and measurement of RuBisCO activity

RuBisCO was isolated from pea leaves according to Tavarini et al. (2016) with minor modifications. 500 mg of pea leaves were ground with liquid nitrogen in mortar and pestle. Then 3 ml of isolation mixture containing 100 mM Tricine − KOH (pH 8.0), 1 mM (EDTA), 1% β − mercaptoethanol (v/v), 1 mM phenylmethylsulfonyl (PMSF), and 5% polyvinylprrolidone (PVP) (w/w of sample fresh weight) was added. Samples where then centrifuged at 12,000 × *g* for 20 min at 4 °C. Protein content in the resulting supernatant was measured by Bradford assay (1976) using bovine serum albumin (Sigma − Aldrich, USA) as a standard.

**Fig. 1:**
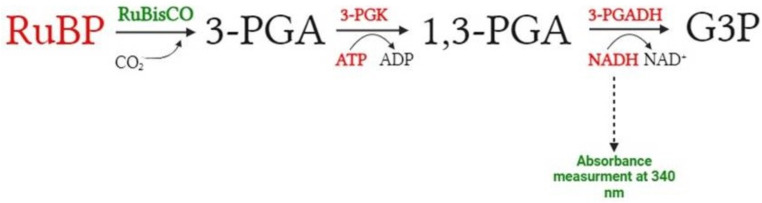
A schematic of the reaction for determining RuBisCO activity

RuBisCO activity was assayed in a reaction mixture containing 100 mM bicine (pH 8.0), 10 mM MgCl_2_, 0.2 mM EDTA, 5 mM dithiothreitol (DTT), 40 mM NaHCO_3_, 4 mM ATP, 0.2 mM NADH, 0.2 mM RuBP, one enzyme unit of 3-phosphoglycerate kinase (PGK) and one enzyme unit of glyceraldehyde 3-phosphate dehydrogenase (3-PGADH). To measure RubisCO activity, a method based on the NADH-linked assay was used. This test is based on RuBP carboxylation and the formation of 3-phosphoglyceric acid (3-PGA). 3-PGA is then converted to 1,3 PGA by 3-phosphoglycerate kinase (PGK) using ATP. 1,3 PGA is then reduced to glyceraldehyde-3-phosphate (G3P) with oxidation of NADH to NAD^+^ by glyceraldehyde 3-phosphate dehydrogenase (3-PGADH). A schematic of the reaction is shown in Fig. 1. 50 µl of enzyme was added to the mixture (final concentration of RuBisCO in reaction mixture was 25 µg/ml) and absorbance spectra were recorded within the wavelength range 310–370 nm for 40 min using the Carry 300 UV Visible Spectrophotometer (Varian, Inc). The reaction temperature was monitored using a laboratory thermometer and held at 25 °C. The reaction was initiated by the addition of RuBP. Single measurement was recorded every 30 s (80 measurements were conducted). RuBisCO activity was calculated according to Sales et al. (2020), using the molar extinction coefficient for NADH, ε = 6220 M/cm. RuBisCO activity was expressed as µmol NADH/ min/mg protein.

### Validation of RuBisCO activity measurement method

In order to validate the method, enzyme activity was measured by maintaining different reaction temperatures: 15 °C, 18 °C, 20 °C, 25 °C and 40 °C using previously described method. Final concentration of RuBisCO in reaction mixture was 25 µg/ml. In addition, different concentrations of RuBisCO were used to measure activity: 100 µg/ml, 50 µg/ml, 25 µg/ml, 12.5 µg/ml, 6.25 µg/ml. Temperature was maintained at 25 °C. Absorbance spectra were recorded within the wavelength range 310–370 nm for 40 min using the Carry 300 UV Visible Spectrophotometer (Varian, Inc) for 40 min. RuBisCO activity was calculated according to Sales et al. (2020), using the molar extinction coefficient for NADH, ε = 6220 M/cm. RuBisCO activity was expressed as µmol NADH/ min/mg protein.

### Statistics

The analyses of plant growth parameters were performed in 10 repetitions. Aminolevulinic acid (ALA) content, aminolevulinic acid dehydrogenase (ALAD) activity, chlorophyll content, RuBisCO content and activity were determined in triplicates. The results were analysed using the Statistica program 11 and a one-way ANOVA test. Differences between groups were analysed using Tukey’s post-hoc test with probability *p* ≤ 0.05.

## Results and discussion

The study examined the effect of tetracycline at concentrations of 5, 50 and 500 mg/kg on pea, with particular attention paid to photosynthetic parameters. The utility of the spectrophotometric method for assessing the activity of RuBisCO was also evaluated in this study. Furthermore, the obtained results were verified through the evaluation of selected photosynthetic parameters, including chlorophyll concentration, aminolevulinic acid (ALA) concentration, aminolevulinic acid dehydrogenase (ALAD) activity, and the content of isolated RuBisCO enzyme.

The first visible symptoms of the action of antibiotics in plants include changes at the morphological level. A general appearance of pea plants is shown in Fig. S1. Fig. S1 presents properly developed seedlings. It is important to note that significant changes in seed germination were observed after treatments with 50 and 500 mg/kg tetracycline (Table 1). The germination rate decreased by 12% and 23%, respectively. Cheong et al. (2020) also demonstrated reduced germination in plants treated with chlorotetracycline. Tetracycline at concentrations of 50 and 500 mg/kg caused a reduction in root length by 6.1 and 9 cm, respectively (Table 1). A decrease in shoot length was observed in plants treated with the highest tetracycline concentration. Xie et al. (2011) also observed a reduction in growth, expressed as a decrease in root and stem length, in wheat treated with higher concentrations of tetracycline (up to 300 mg/l). The authors state that the root is more sensitive to tetracycline action than the shoot, which was confirmed by our results. A change in the color of photosynthetic organs is the first symptom of tetracycline action on the photosynthetic apparatus (Krupka et al. 2022). At the morphological level, the effect of tetracycline in plants may also be expressed as a reduction in leaf area. The total leaf surface area of pea plants growing in soil contaminated with tetracycline at concentrations of 50 and 500 mg/kg decreased by 7.6% and 10%, respectively, compared to the control (Table 1).

**Table 1:** Growth parameters of pea grown on soil with 0, 5, 50 and 500 mg/kg tetracycline addition. Means marked with different letters differ significantly (*p* ≤ 0.05) between groups (ANOVA, Tukey HSD test)

Tetracycline concentration in soil [mg/kg]	Germination [%]	Root length [cm]	Shoot length [cm]	Total leaf area [cm^2^]
**0**	98 ± 0.3 **a**	16.5 ± 2.3 **a**	14.5 ± 1.4 **a**	136.0 ± 7.8 **a**
**5**	98 ± 0.3 **a**	15.9 ± 2.5 **a**	14.1 ± 1.1 **a**	134.1 ± 5.6 **a**
**50**	88 ± 0.4 **b**	10.4 ± 1.5 **b**	13.1 ± 1.0 **a**	125.7 ± 5.2 **b**
**500**	77 ± 0.4 **c**	9.5 ± 0.9 **b**	12.8 ± 0.9 **b**	122.3 ± 4.2 **b**

Changes in the morphology of photosynthetic organs in plants cultivated in antibiotic-contaminated soils result from alterations in the photosynthetic apparatus. One of the key factors determining proper plant and organ growth is chlorophyll. The direct mechanism of chlorophyll degradation by tetracycline was demonstrated by Rydzyński et al. (2019). The authors showed that the degradation product of chlorophyll resulting from the action of tetracycline is pheophytin, and the mechanism of this process involves the direct removal of the Mg^2+^ ion from chlorophyll by tetracycline. The removed magnesium binds to the tetracycline molecule at two sites - the BCD ring and the A ring, depending on the magnesium concentration (Rydzyński et al. 2019).

**Fig. 2:**
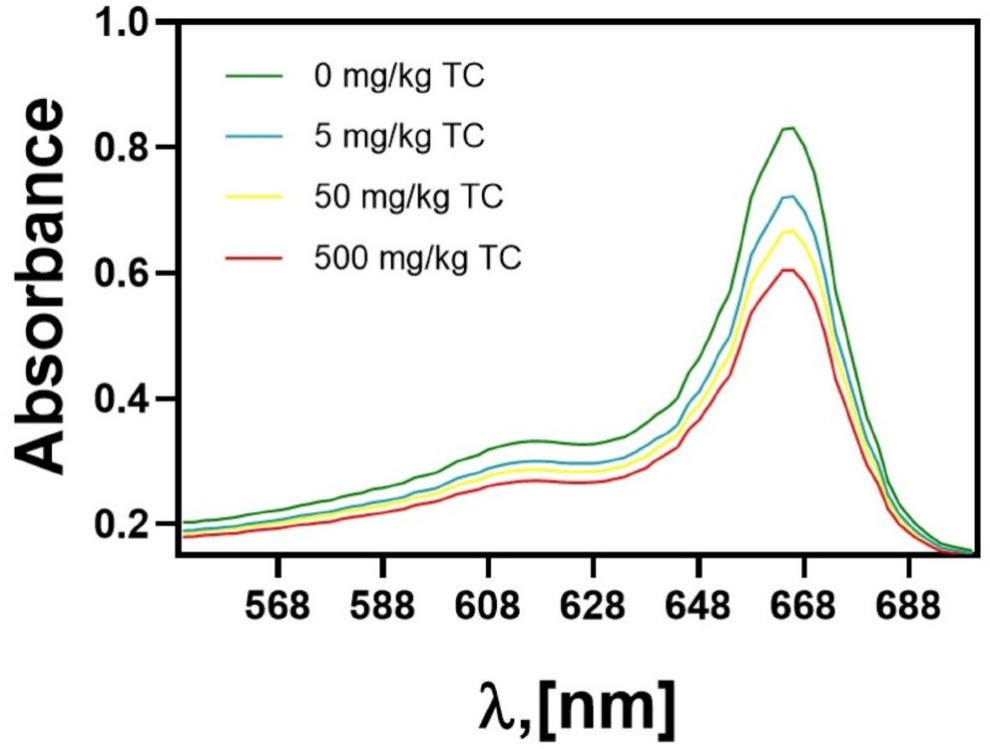
Absorption spectra of chlorophyll isolated from pea grown on soil

Our results indicate that the lowest tetracycline concentration (5 mg/kg) led to a decrease in chlorophyll a absorbance at λ = 665 nm from A = 0.83 to A = 0.72 (Fig. 2) (a 17% decrease in chlorophyll concentration in the plant) (Table 2). These findings clearly demonstrate that even low concentrations of tetracycline, as sometimes detected in the environment, cause chlorophyll degradation. Increasing the tetracycline concentration 500 mg/kg resulted in a decrease in absorbance to A_665_ = 0.6, respectively (Fig. 2), and thus a reduction in chlorophyll a concentration by 20% and 28% (Table 2).

**Table 2:** Chlorophyll a, b, total chlorophyll content (mg/cm^2^) and chlorophyll a/b ration in pea grown on soil with 0, 5, 50 and 500 mg/kg tetracycline addition. Means marked with different letters differ significantly (*p* ≤ 0.05) between groups (ANOVA, Tukey HSD test)

Tetracycline concentration in soil [mg/kg]	Chlorophyll a content [µg/cm^2^]	Chlorophyll b content [µg/cm^2^]	Total chlorophyll [µg/cm^2^]	Chlorphyll a/b ratio
**0**	0.47 ± 0.01 **a**	0.26 ± 0.006 **a**	0.73 ± 0.012 **a**	1.79 ± 0.056
**5**	0.39 ± 0.006 **b**	0.24 ± 0.006 **b**	0.63 ± 0.01 **b**	1.67 ± 0.035
**50**	0.39 ± 0.006 **b**	0.24 ± 0.012 **b**	0.63 ± 0.015 **b**	1.62 ± 0.104
**500**	0.34 ± 0.015 **c**	0.25 ± 0.006 **b**	0.60 ± 0.01 **c**	1.37 ± 0.089

Some studies indicate that tetracycline can block the synthesis of both chlorophyll a and b by inhibiting enzyme biosynthesis. A decrease in chlorophyll b content in tetracycline-treated *Lemna minor* was demonstrated by Baciak et al. (2016). However, our results indicate a slight decrease in chlorophyll b concentration in peas (Table 2). It can therefore be concluded that chlorophyll a is much more sensitive to tetracycline-induced degradation, than chlorophyll b. The chlorophyll a/b ratio is a good parameter for assessing the state of the photosynthetic apparatus in plants. Any disturbances of this parameter may indicate damage of the photosynthetic apparatus (Krupka et al. 2022). Our results indicate that tetracycline in a concentration range 5–500 mg/kg caused a disturbance in the chlorophyll a/b ratio (Table 2).

**Fig. 3:**
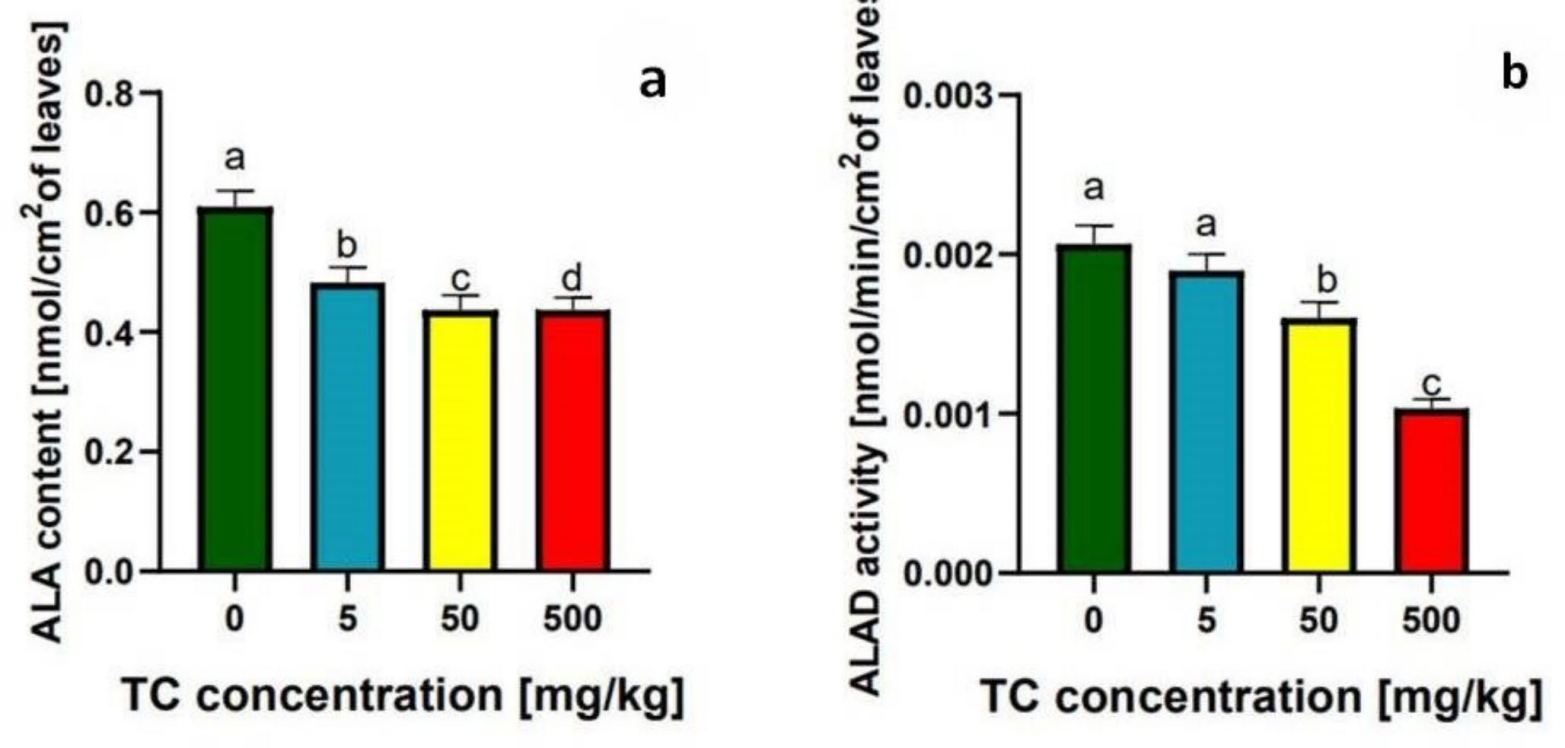
δ-Aminolevulinic acid (ALA) content [nmol/cm^2^ of leaves] (**a**) and acid dehydrogenase (ALAD) activity [nmol PGB/min/mg protein/ cm^2^ of leaves] (**b**) in peas grown on soil without tetracycline and 5, 50 500 mg/kg tetracycline addition. Means marked with different letters differ significantly (*p* ≤ 0.05) between groups (ANOVA, Tukey HSD test)

To demonstrate at which stage tetracycline may interfere with the photosynthesis, the activity of an enzyme involved in chlorophyll biosynthesis (ALAD) was examined. However, our results indicate that despite the inhibition of ALAD activity (Fig. 3a) in plants treated with higher tetracycline, there was also a reduction in the concentration of ALA (Fig. 3b). Similar results were obtained by Liu et al. (2014) when treating *Selenastrum capricornutum* with antibiotics. The authors pointed out that erythromycin, as an antibiotic inhibiting bacterial protein synthesis, exhibits high toxicity towards chlorophyll precursors, even at low concentrations. As a mechanism of erythromycin’s toxicity towards chlorophyll precursors, the authors identified the inhibition of chloroplast gene expression, and subsequently, indirectly, nuclear genes responsible for chlorophyll biosynthesis (Liu et al. 2014). Tetracycline, as an antibiotic inhibiting protein synthesis, may also exhibit a similar mechanism of toxicity. However, further research is needed to address this issue.

RuBisCO is the most widely occurring plant protein, consisting of 8 large and 8 small subunits (Abdulbaki et al. 2022). The small subunits are encoded by the nuclear gene family rbcS, while the large subunits are chloroplast-encoded, by the rbcL gene (Wostrikoff and Stern 2007). Although the impact of pharmaceuticals on the structure and activity of the RuBisCO enzyme is only partially understood, Liu et al. (2014) demonstrated reduced expression of the rbcL gene in the chloroplasts of *Microcystis aeruginosa* algae treated with antibiotics. Similar results were obtained by Yoon et al. (2020) when treating *Brassica campestris* with erythromycin at a concentration of 5 mg/kg. As an antibiotic inhibiting protein synthesis, erythromycin reduced the expression level of proteins involved in photosynthesis, including the large subunit of RuBisCO, leading to a decrease in its content. The down regulation of the rbcL gene was identified as the cause of the reduced protein content (Yoon et al. 2020). Tetracycline, as an antibiotic inhibiting protein synthesis, likely exhibits a similar mechanism of action.

**Fig. 4:**
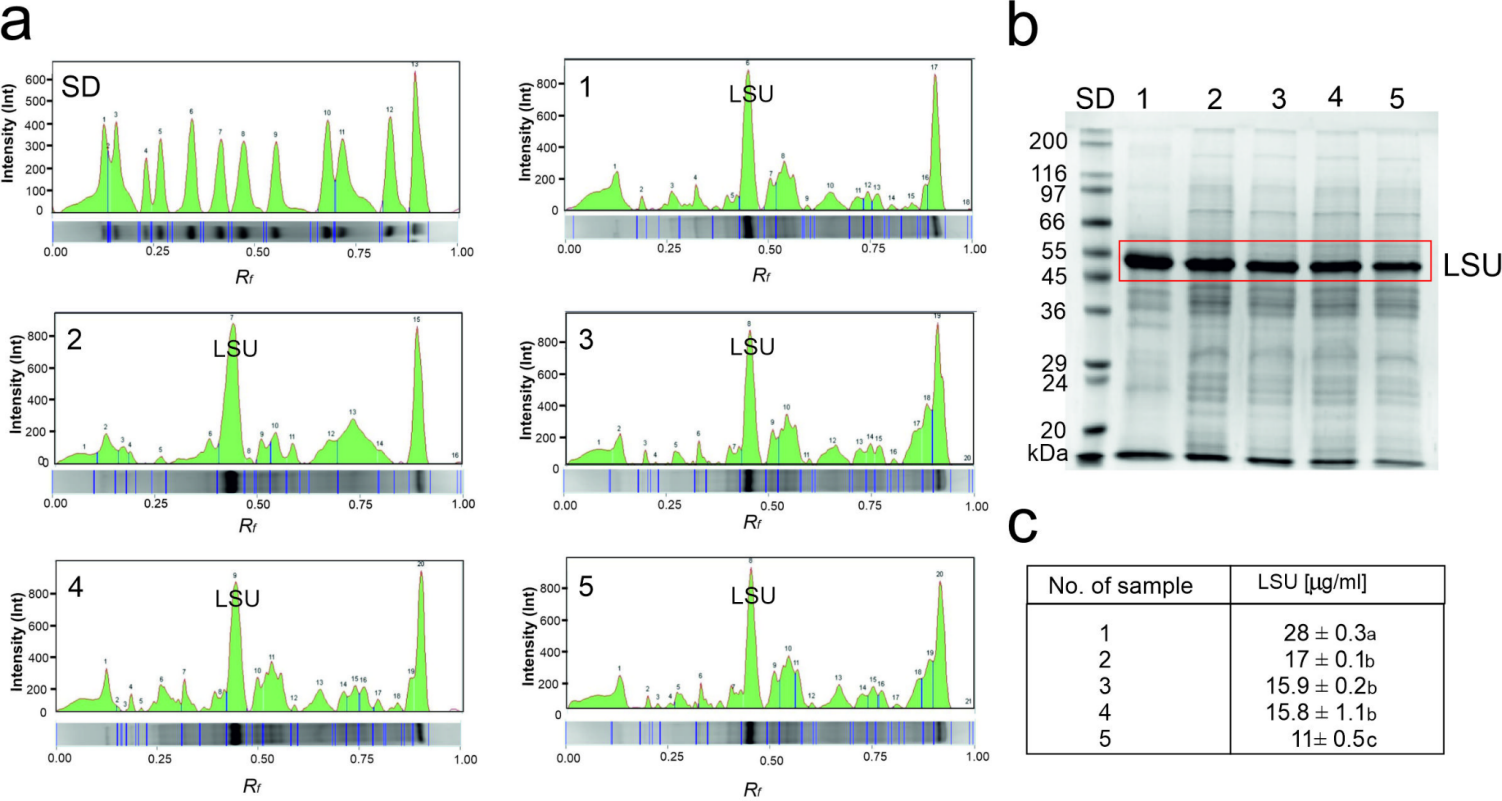
**a** - Intensity of individual proteins; **b** - SDS – PAGE protein pattern and large subunit concentration (LSU) of RuBisCO; **c** – Content of large subunit concentration (LSU); SD – standard of protein, 1 – commercial RuBisCO, 2 – RuBisCO isolated from pea grown on soil without tetracycline, 3 – RuBisCO isolated from pea grown on soil with 5 mg/kg tetracycline addition, 4 – RuBisCO isolated from pea grown on soil with 50 mg/kg tetracycline addition, 5 – RuBisCO isolated from pea grown on soil with 500 mg/kg tetracycline addition

**Fig. 5:**
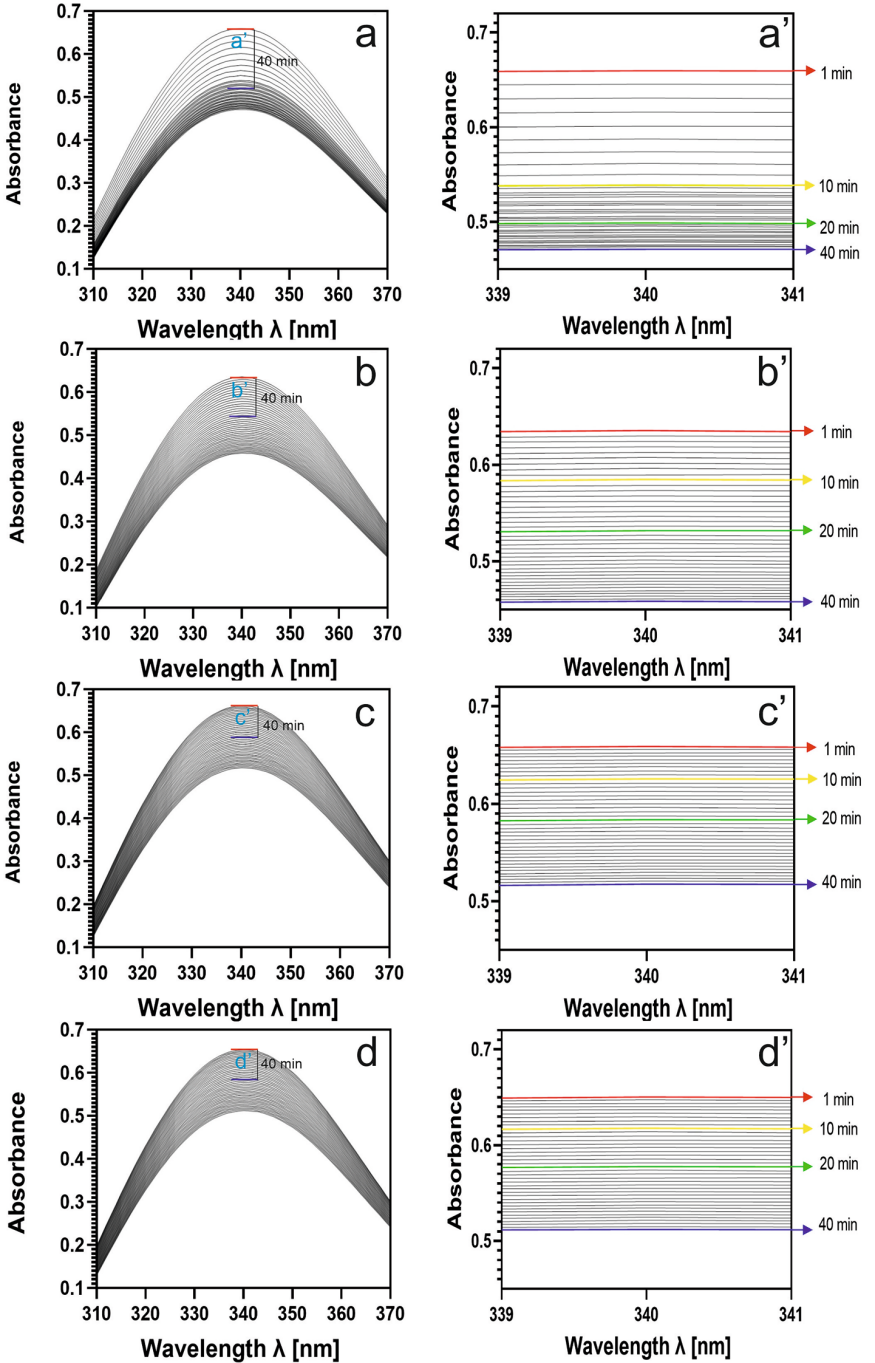
Absorption spectra of NADH (80 measurements) in the wavelength range of 310–370 nm for determining RuBisCO activity from peas grown on soil contaminated with tetracycline at doses of 0 (**a**), 5 (**b**), 50 (**c**), and 500 (**d**) mg/kg. The rectangles marked on these figures refer to NADH absorption in the narrow range of 339 to 341 nm, whose data are presented separately in panels **a**’, **b**’, **c**’, and **d**’. Absorption measurements were taken over 40 min

The content of the large subunit of RuBisCO (LSU) in plants treated with tetracycline was assessed in this work using SDS-PAGE electrophoresis (Fig. 4). To properly assess the quality of the isolated enzyme from peas, we conducted an SDS-PAGE analysis (Fig. 4b), whose quality we evaluated by calculating the area of the obtained electrophoretic bands after conversion to peaks (Fig. 4a). The most abundant leaf protein fraction was in the 50–55 kDa mass range (Fig. 5), with a content of 17 µg/ml (Table 2). This identification of this protein fraction as the large subunit of RuBisCO was confirmed by comparison to a commercial enzyme. In the commercially purchased RuBisCO, the area of the LSU was 1802, and for the LSU isolated after applying tetracycline at concentrations of 0, 5, 50, and 500 mg/kg, the areas were 1387, 1294, 1228, and 896, respectively. However, the total area of all proteins (calculated from Fig. 4a) isolated was approximately 4,000,000 area units for all five variants, including the commercial RuBisCO. The analyzed protein was used at the same concentration for all variants, which fully confirms the good quality of the isolated enzyme. The application of tetracycline at concentrations of 5, 50, and 500 mg/kg resulted in a decrease in LSU content by 6.5%, 11%, and 35%, respectively, compared to the control (Fig. 4c). Cohen et al. (2005) indicate that the removal of one subunit can influence the expression of other subunits in the complex, thereby affecting the enzyme’s activity.

The activity of RuBisCO is a crucial factor determining the efficiency of the photosynthesis process. Under abiotic stress conditions, one of the factors leading to reduced enzyme activity is the degradation of RuBisCO activase. RuBisCO activase acts as a chaperone responsible for changing the enzyme’s conformation, thereby regulating its function (Waheeda et al. 2023). Margas et al. (2019) demonstrated a decreased concentration of RuBisCO activase in pea seedlings treated with tetracycline; however, the impact of tetracycline on the activity of this enzyme is not well-documented. Therefore, both the decrease in LSU content and the degradation of activase may affect RuBisCO activity. Our current results indicate, however, that tetracycline caused a decrease in RuBisCO activity more pronounced than the drop in RuBisCo quantity in pea leaves. The application of an antibiotic in the soil at a concentration of 5 mg/kg resulted in a 19% decrease in enzyme activity compared to the control (Table 3). Increasing the concentration of tetracycline to 50 and 500 mg/kg resulted in a decrease in RuBisCO activity by 37% and 47% (Table 3). Figure 5a-d shows the decrease in absorbance over time (40 min). The decrease in absorbance at 340 nm over time indicates the progression of the reaction catalysed by RuBisCO. A decrease in absorbance at λ = 340 nm from A = 0.672 to A = 0.471 was observed in control pea, indicating high enzymatic activity. After treatment with tetracycline, the decrease in absorbance was slower, suggesting an inhibitory effect of tetracycline on RuBisCO activity. For pea treated with 5 mg/kg tetracycline, a decrease in absorbance at λ = 340 nm from A = 0.636 to A = 0.459 was observed (Fig. 5b). Increasing the concentration of tetracycline to 50 mg/kg resulted in a decrease in absorbance from A = 0.662 to A = 0.518. The highest tetracycline concentration caused a decrease in absorbance at λ = 340 nm from A = 0.653 to A = 0.511 (Fig. 5d). Therefore, even low concentrations of tetracycline, as found in the environment, lead to reduced RuBisCO activity, thereby influencing a decrease in photosynthetic efficiency. The linear decrease in absorbance at 340 nm is shown in Fig. 5a’-d’. The graph can be used to validate the assay method by demonstrating a consistent linear relationship under controlled conditions. Smaller distances between lines may suggest that tetracycline inhibits the enzyme activity (Fig. 5b’–d’). The NADH absorption between two measurements for RuBisCO isolated from plants grown on soil uncontaminated with tetracycline was 0.02, whereas for plants from soil contaminated with the highest dose of tetracycline, the decrease in absorption between two measurements was only 0.00085, which we observe clearly as a densification of the lines. Comparing the control samples (Fig. 5a’) with those treated with tetracycline, we observe a significant reduction in the rate of absorbance decrease in the treated samples, indicating an inhibitory effect of tetracycline on RuBisCO activity.

**Table 3:** Activity [µmol NADH/min/mg protein] of RuBisCO isolated from pea grown on soil with 0, 5, 50 and 500 mg tetracycline/kg of soil after 10 and 20 min. RuBisCO activity were measured using NADH-linked microtiter plate-based assay: GAPDH–GlyPDH. Means marked with different letters differ significantly (*p* ≤ 0.05) between groups (ANOVA, Tukey HSD test)

Tetracycline mg/kg	Activity of RuBisCO [µmol NADH/min/mg protein]		
	10 min	20 min	Linear decline in absorbance
**0**	0.234 **a**	0.181 **a**	0.234 **a**
**5**	0.098 **b**	0.099 **b**	0.099 **b**
**50**	0.064 **c**	0.072 **c**	0.072 **c**
**500**	0.062 **c**	0.069 **c**	0.070 **c**

In this study, RuBisCO activity was investigated using a spectrophotometric method, employing absorbance spectrum measurements. Spectrophotometric method can be applied to this enzyme as an alternative to the more prevalent radioactive methods utilizing ^14^C to monitor the rate of product formation. Although the radioactive method is considered accurate and specific, its application requires appropriate training and adherence to strict safety regulations (Koussevitzky et al. 2007). The method used in this study provides a rapid and effective alternative for assessing RuBisCO enzyme activity. Most studies utilizing the spectrophotometric method for RuBisCO activity recommend measuring the reaction for 10 min. To determine the reaction time, measurements were taken every 30 s, resulting in 80 measurements. Based on the absorbance spectra measurements (Fig. 5a-d), it was demonstrated that a 10-minute measurement time is too short to assess RuBisCO activity accurately. The enzymatic reaction concluded after 20 min of measurement (Fig. 5a-d). Therefore, using a 10-minute activity measurement may lead to obtaining inaccurate results (Table 3).

Validation of the method for the determination of RuBisCO activity was performed in this study. According to Sales et al. (2020), the concentration of unpurified RuBisCO in the reaction should vary between 10 and 40 µg/ml. Concentrations exceeding this range may limit the sensitivity of NADH-linked assays (Sales et al. 2020). Our results indicate that the application of higher enzyme concentrations in the reaction (50–100 µg/ml) results in lower enzyme activity (Table 4). The use of lower concentrations – 6.25 µg/ml – 12.5 µg/ml – does not allow for the determination of enzymatic activity (Table 4). Another factor significantly affecting the efficiency of the reaction is temperature. Our results indicate that the highest activity of RuBisCO is achieved at a temperature of 25 °C. Lower and higher values result in a significant decrease in the efficiency of the reaction (Table 4).

**Table 4:** RuBisCO activity depending on temperature (15 °C, 18 °C, 20 °C, 25 °C, 40 °C) and RuBisCO concentration (100 µg/ml, 50 µg/ml, 25 µg/ml, 12.5 µg/ml, 6.25 µg/ml). Means marked with different letters differ significantly (*p* ≤ 0.05) between groups (ANOVA, Tukey HSD test)

**Temperature**					
	Activity of RuBisCO [µmol NADH/min/mg protein]			Linear decline in absorbance	
**15 °C**	0.100			0.104	
**18 °C**	0.145			0.150	
**20 °C**	0.164			0.170	
**25 °C**	0.236			0.200	
**40 °C**	0.096			0.100	
**RuBisCO concentration**					
	Activity of RuBisCO [µmol NADH/min/mg protein]			Linear decline in absorbance	
**100 µg/ml**	0.027			0.111	
**50 µg/ml**	0.099			0.207	
**25 µg/ml**	0.234			0.200	
**12.5 µg/ml**	No activity			0.004	
**6.25 µg/ml**	No activity			0	

## Electronic supplementary material

Below is the link to the electronic supplementary material.

Supplementary Material 1

## Data Availability

The raw data will be available at the University of Warmia and Mazury web page.https://bazawiedzy.uwm.edu.pl/info/researchdata/UWMd130cd42364e484d90bc3af029dba69c?r=researchdata&ps=20&tab=&title=Szczeg%25C3%25B3%25C5%2582y%2Brekordu%2B%25E2%2580%2593%2BDane%2Bbadawcze%2B%25E2%2580%2593%2BUniwersytet%2BWarmi%25C5%2584sko-Mazurski&lang=pl
