# Supplementary material for: Critical stages in pea photosynthesis impaired by tetracycline as an environmental contaminant
Source: J Plant Res. 2024 Sep 21;137(6):1049–59. doi: 10.1007/s10265-024-01580-x (PMC11525401; doi:10.1007/s10265-024-01580-x)
Supplement: Supplementary file 1 — Supplementary Material 1 [file 10265_2024_1580_MOESM1_ESM.pdf]

Critical stages in pea photosynthesis impaired by tetracycline as an environmental contaminant

Magdalena Krupka, Dariusz J. Michalczyk, Agnieszka I. Piotrowicz-Cieślak*
Department of Plant Physiology, Genetics and Biotechnology, University of Warmia and Mazury in Olsztyn
*Correspondence: acieslak@uwm.edu.pl

Supplementary material

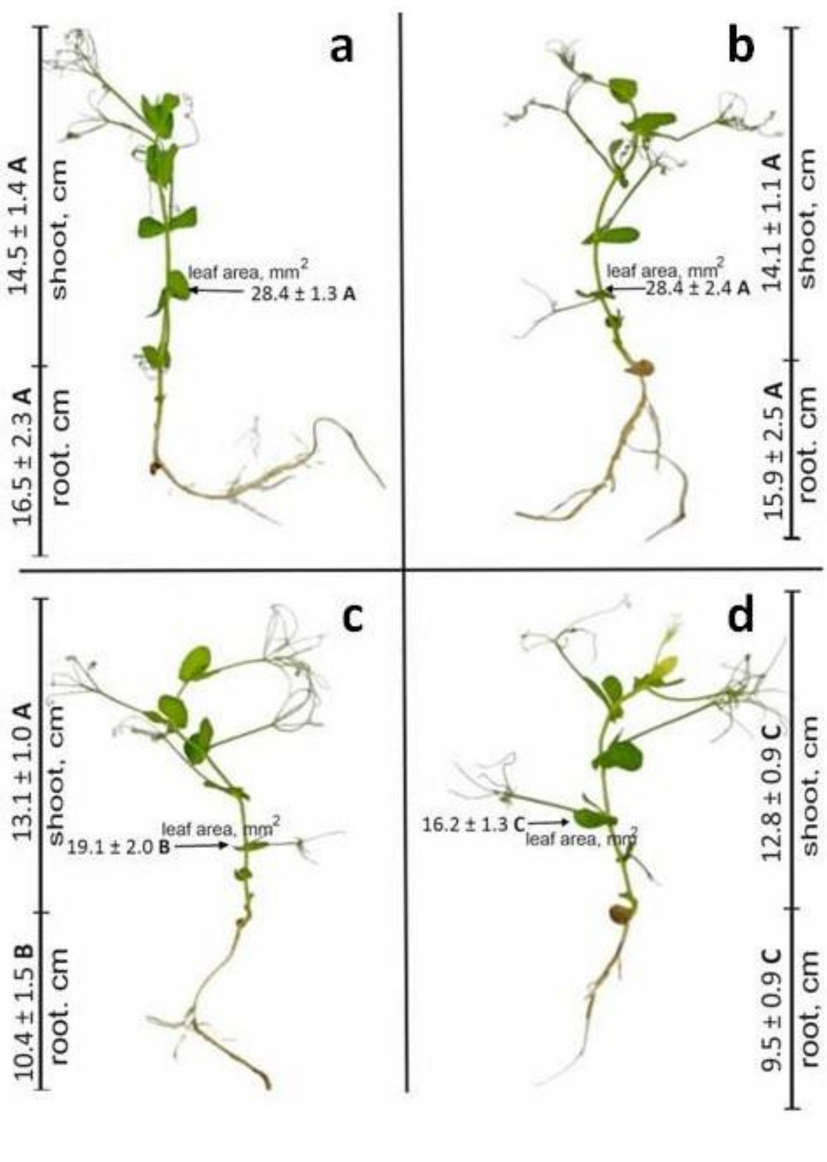

Figure S1. General appearance of pea (a- pea grown on soil without tetracycline, b- pea grown on soil with 5 mg/kg tetracycline addition, c- pea grown on soil with 50 mg/kg tetracycline addition, d- pea grown on soil with 500 mg/kg tetracycline addition).
